# Acute Right Ventricular Failure in the Medical ICU

**DOI:** 10.1007/s00408-025-00862-y

**Published:** 2025-12-08

**Authors:** Amos E. Dodi, Mark Jacobs

**Affiliations:** Division of Critical Care Medicine, Department of Medicine, Montefiore Medical Center, Albert Einstein College of Medicine, 111 East 210 Street, Bronx, NY 10467 USA

**Keywords:** Right ventricular failure, Right heart failure, Pulmonary embolism, Pulmonary hypertension, Critical care, Intensive care unit

## Abstract

Right ventricular failure (RVF) is a complex clinical syndrome resulting from anatomical and physiological dysfunction of the right ventricle, marked by insufficient cardiac output state, elevated filling pressures, and elevated central venous pressures. Historically, acute RVF in the medical intensive care unit (MICU) has posed significant diagnostic and therapeutic challenges, often leading to poor patient outcomes and increased healthcare utilization. RVF is a pervasive and critically underdiagnosed condition in MICUs, often masked by nonspecific symptoms and overlooked in favor of left-sided pathology, despite its profound impact on patient outcomes and mortality. This difficulty stems from a limited understanding of its underlying mechanisms and a lack of high-quality evidence to guide management in critical care settings. Effective care for RVF demands early recognition, precise identification of the underlying etiology, and prompt, targeted interventions. Intensivists must possess comprehensive knowledge and a diverse skill set to navigate these complexities and address unforeseen complications. Over the past two decades, advancements in diagnostic and therapeutic technologies have transformed the approach to RVF, driving significant progress in the field. This review explores the historical evolution, pathophysiology, clinical presentation, and contemporary management strategies for RVF in the MICU.

## Introduction

Right ventricular failure is a complex and multifaceted clinical syndrome frequently encountered in the medical intensive care unit, yet it remains less commonly diagnosed and studied compared to cardiogenic shock from acute left ventricular (LV) failure, which is widely discussed in the medical literature. The challenges in diagnosing and treating RVF arise from the intricate interconnection between the right ventricle’s structure and function, compounded by a lack of clear definitions and difficulties in distinguishing RVF from RV dysfunction. In 2018, an American Thoracic Society working group proposed that “RVF is a complex clinical syndrome characterized by insufficient blood delivery from the RV, accompanied by elevated systemic venous pressure at rest or during exercise” [1]. Due to the limited evidence specific to RVF, many trials referenced in this review, particularly in the management section, were conducted in the broader heart failure (HF) population, likely including patients both with and without RVF, yet many management strategies apply to both.

The interplay between the hemodynamics of the RV and the pulmonary vasculature is an essential foundation of optimal circulation during critical illness and alterations in these function-structure relationships can be detrimental. The inability of the RV to maintain venous return necessary for adequate cardiac output (CO), in the presence of elevated venous pressure, is the pivotal mechanism leading to poor outcomes [2]. Common ICU interventions and many disease processes are exacerbated and adversely impacted by RVF. Among these are positive pressure ventilation (PPV), septic shock, pulmonary thromboembolism (PE), decompensated pulmonary hypertension (PH) and the numerous conditions arising from acutely increased pulmonary vascular resistance (PVR), such as: hypoxia, acidosis, pneumonia and ARDS. The introduction of diagnostic tools such as point-of-care ultrasound (POCUS); facilitating non-invasive and rapid real-time evaluation at the bedside, revolutionary new therapeutics to manage pulmonary hypertension (PH), and the multitude of temporary mechanical circulatory devices (MCD) allows for more accurate diagnosis, more treatment options and improved outcomes. The purpose of this review is to describe the complex background of this disease entity and assist the intensivist with the correct diagnosis and management employing the most recent advances in the field. Whenever feasible, we have sought to offer a practical framework and actionable advice for clinicians, drawing on our experience and the available published data. Due to the limited number of high-quality studies, these recommendations are not intended to serve as formal clinical practice guidelines.

## Epidemiology

**Fig. 1:**
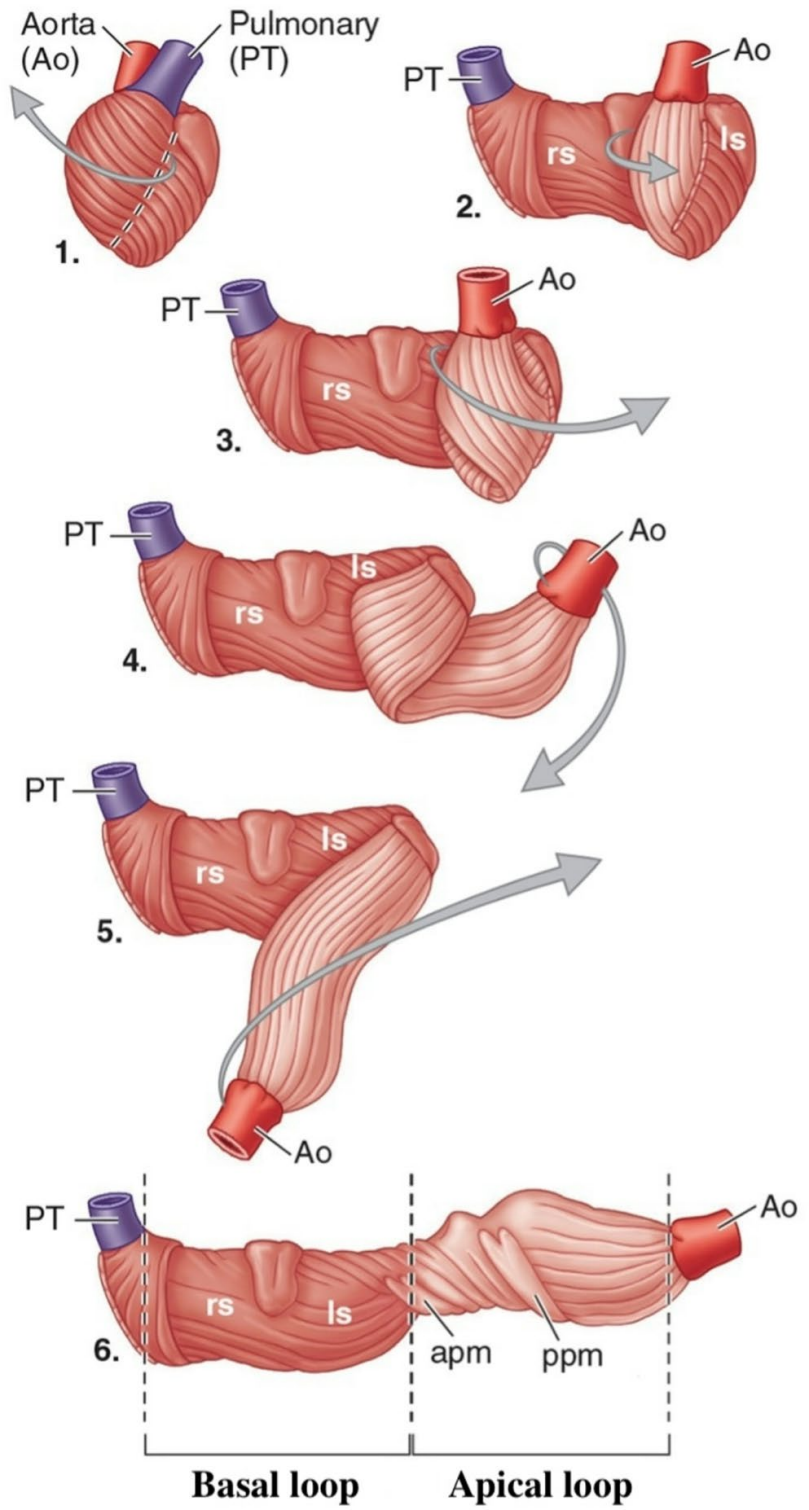
The double loop helical arrangement of the myocardium. Incision of the myocardial surface along the anterior interventricular groove (dashed line in 1) and reflected starting at the pulmonary trunk (PT; 2). The ventricular myocardial band is progressively unraveled (3–6). A nearly horizontal band forms the outer basal loop (dark muscle; 6), which constitutes the outer wall of the RV (right segment, rs) and the external layer of the outer wall of the LV (left segment, ls). The deeper apical loop (light muscle; 6) constitutes the internal layer of the outer wall of the left ventricle. Its intersecting fibers form a double-layered interventricular septum. Rather than merely collapsing inward, the sequential contraction of the myocardial band enables the ventricles to draw and propel blood in a coordinated fashion. apm, anterior papillary muscles; ppm, posterior papillary muscles. Adapted with permission from Moore's clinically oriented anatomy, 2023 [11]

There is limited data about the prevalence of RVF in the critical care setting and since multiple major disease states lead to RVF, it is likely underreported [3]. RVF accounts for up to 2.2% of acute heart failure admissions but is present in up to one-fifth of acute LV failure admissions [4] and is responsible for 5–17% of in-hospital mortality [5]. 48% of patients with acute HFrEF and 20–40% of patients with HFpEF have RV dysfunction [6]. A study by Ghio et al. [7] showed that in patients with HFrEF, the combination of high Pulmonary Artery Pressure (PAP) and low RV ejection fraction (RVEF) leads to 50% lower survival in 70 months compared with HFrEF patients with either condition or none (Fig. 1).

## Anatomy

**Fig. 2:**
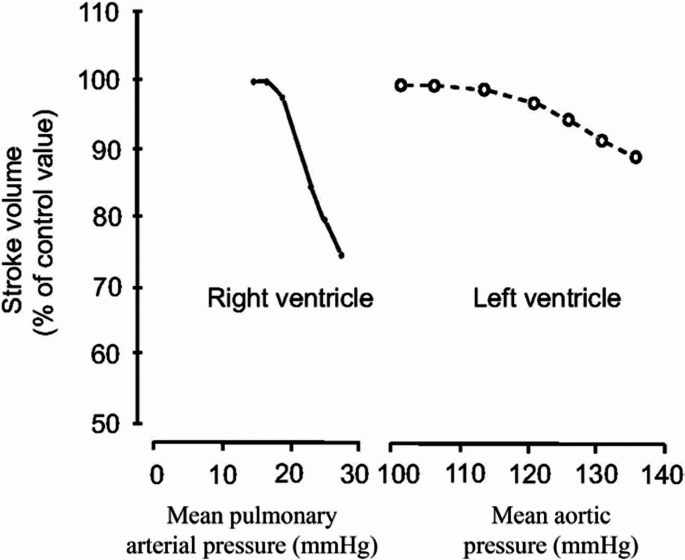
Comparison of the RV and LV response to experimental increase in afterload. Noted is a significant reduction in SV with a small increase in mean pulmonary arterial pressure compared to a modest reduction in LV SV when acted upon by similar increments. Reprinted with permission from Konstam et al., [2] adapted from Braunwald E. Pathophysiology of heart failure. In: Braunwald E, ed. Heart Disease: A Textbook of Cardiovascular Medicine. Philadelphia, PA: WB Saunders; 1980:453–471

**Fig. 3:**
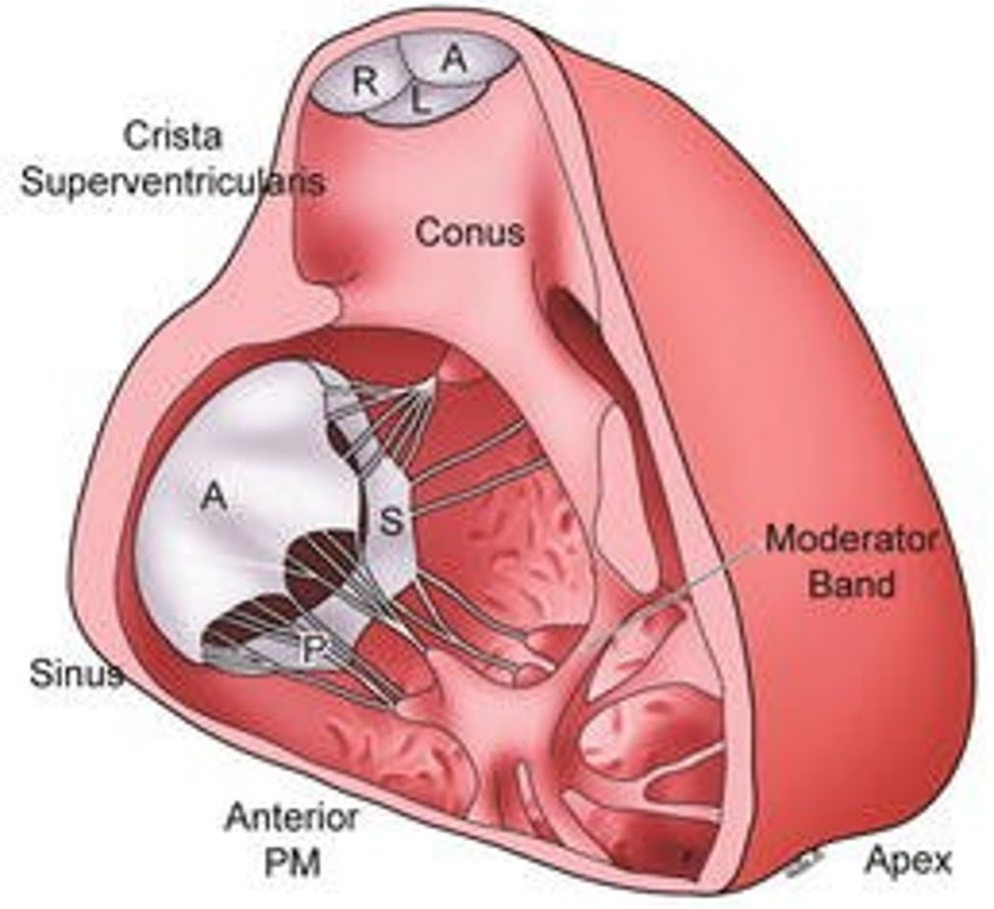
Illustration of the anatomy of the right ventricle, highlighting the Crista Supraventricularis and the Moderator Band. Together, these facilitate efficient RV contraction and blood ejection by directing blood flow to the pulmonary outflow tract and synchronizing and reinforcing RV contraction respectively. Reprinted with permission from Perioperative Two-Dimensional Transesophageal Echocardiography, 2018 [21]

The RV cavity has a complex geometrical structure; its chamber consists of an inlet and an outlet portioned by the crista supraventricularis (Fig. 3). Its medial wall is the intraventricular septum (IVS), a double-layered structure composed of muscle fibers from both the LV and the RV, with the LV fibers being the dominant contributors to IVS contraction, while its lateral wall is a free wall. The widely debated theory of the Ventricular Myocardial Band of Torrent-Guasp (TG) provides a possible explanation for the ability of the heart to generate significant work despite sarcomere length shortening of merely 15% [8]. This model suggests that the heart's muscle fibers are arranged in a continuous, helical band that wraps around the ventricles, rather than the traditional view of layered, separate muscle groups. After performing hundreds of dissections, TG concluded that the ventricles are composed of a continuous band of myocardium folded on itself in a helix formation (Fig. 2) [9]. This proposed structure is composed of two loops; the basal, divided into the right and left segments, and the apical, composed of the descendent and ascendant segments. The apical loop gives rise to the IVS and the LV and has circumferential muscle orientation, while the basal loop gives rise to the RV free wall and has transverse muscle orientation. The result of sarcomere shortening in the apical loop is a twisting, piston-like torsional movement impelling blood forcefully out from the apex towards the base, while contraction of the basal loop leads to radial compression. Whether TG’s theory is completely correct or not, it supports the observation that preserved vertical contraction of the IVS is a pivotal component in the ability of the RV to maintain adequate CO and a major contributor to RVEF. The development of RVF is in large part due to impairment of septum twisting by the counterforce of increased afterload [10]. Abnormal septum function serves as a critical indicator, signaling an imbalance in tissue load between the right and left ventricles.

## Pathophysiology

The RV integrates cardiac preload, afterload, rhythm, contractility, pericardial constraint, and intraventricular interactions. It does the same amount of work as the LV requiring only 20% of the energy with less than half the wall thickness and 25% of the muscle mass of the LV, yet its output is similar to that of the LV [12]. This is possible because systemic vascular resistance (SVR) is 10 times that of the PVR [13]. Working against the highly compliant, low-resistance pulmonary circulation allows the RV to move a relatively high volume with low pressure (Fig. 3). Furthermore, due to the RV's lower operating pressures, its isovolumic contraction and relaxation phases are briefer and less pronounced, enabling blood flow into the pulmonary artery even during early diastole [14]. However, unlike the LV, the RV responds poorly to high pressures and primarily dilates in response to increased afterload [15]. The RV has a significant capacity to adapt to chronic increases in PVR with dilation and hypertrophy as opposed to its poor ability to adapt to acute changes. When taken to the extreme, these changes can also lead to worsening TR from tricuspid annular dilatation and myocardial ischemia from extreme hypertrophy [16].

Under normal conditions, the pulmonary circulation adapts to increased volume without a significant increase in pressure. This is possible through recruitment of under-perfused capillary beds in the upper zones of the lung where the perfusion pressure is lowest due to the effects of gravity [17]. One of the most striking phenomena demonstrating this is how the remaining lung of pneumonectomy patients can take the entire pulmonary blood flow, at rest, without an increase in pulmonary artery pressure [18]. Damiano et al. [19] electrically isolated the right and left ventricles in canine hearts and measured their respective pressures and flows. When RV afterload was normal, pacing the LV alone fully maintained RV pressure and PA flow. In contrast, pacing the isolated RV had almost no effect on LV or aortic pressures. These findings demonstrated that, under normal conditions, the LV can sustain right-heart output, whereas the RV contributes little to left-heart performance. Damiano concluded that the IVS is electrically activated as part of the LV myocardium and accounts for more than 60% of total RVEF. These findings are supported by other experiments showing that electrocautery and patch replacement do not alter RV function when the IVS is intact [8, 20]. They also provide the physiological basis for the effective use of inotropes in the treatment of RVF through the activation of β1 receptors in the LV portion of the IVS.

While the aortic and mitral valves share a fibrous tissue structure called the Aortic Mitral Curtain, the Crista Supraventricularis (CS) is a more flexible muscular bridge separating the RV inflow and outflow tracts (Fig. 3) and sharing muscle fibers with both the IVS and the RV free wall [16]. During systole, the CS facilitates the contraction of the orifice of the tricuspid valve (TV) while pulling the RV free wall inwards towards the IVS. As the RV dilates from volume overload, the CS elongates resulting in functional tricuspid regurgitation (TR) and decreased RV free wall radial contraction leading to further increased RV volume overload and the creation of a vicious cycle.

**Fig. 4:**
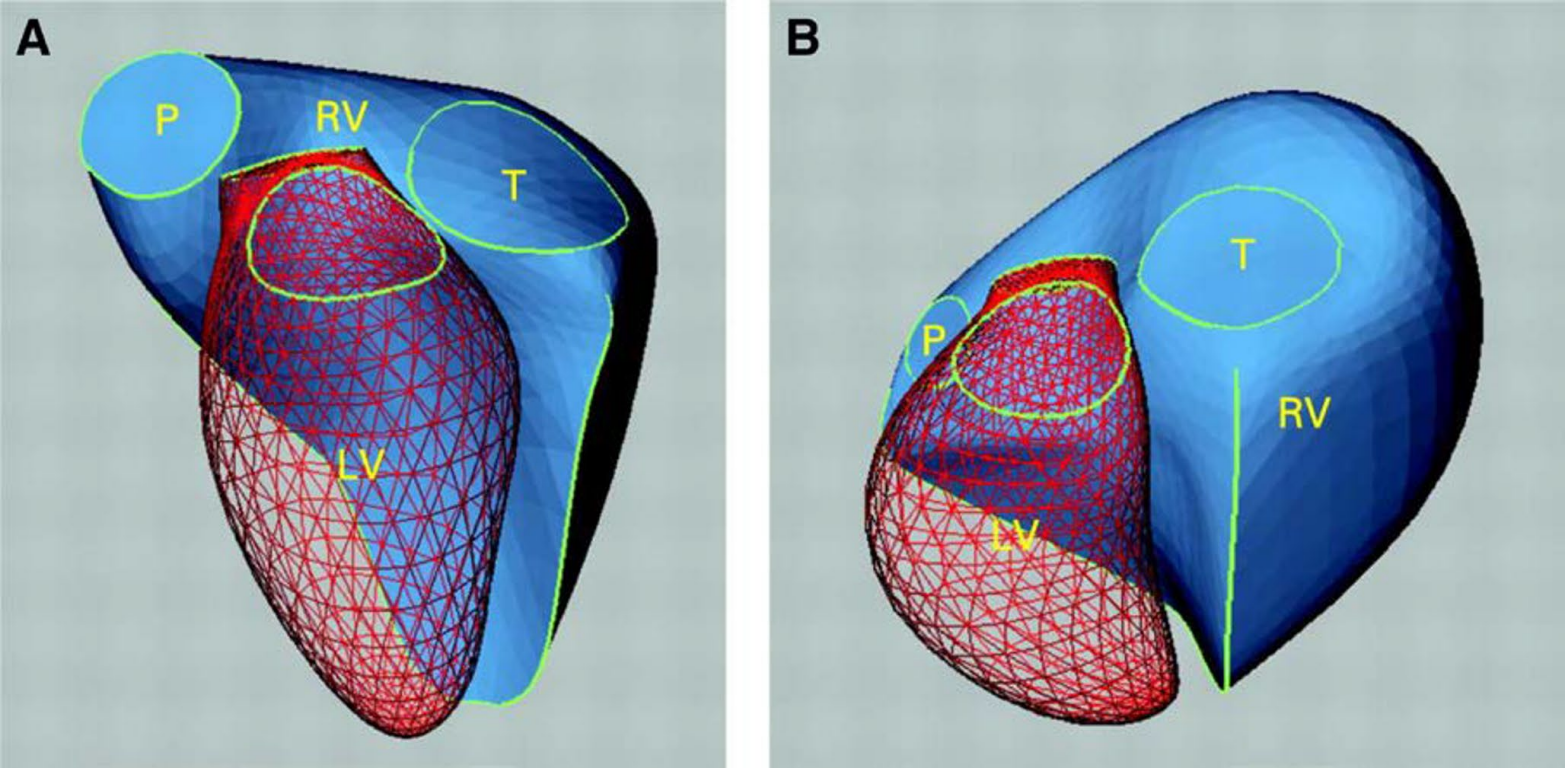
Three dimensional reconstructions of the right ventricle (RV) illustrating its complex shape in a normal subject (**A**). RV remodeling in diseased hearts can result in profound shape change, as in this patient (**B**) with dilated RV due to severe pulmonary regurgitation following repair of tetralogy of Fallot. The mesh surface is the left ventricle. LV, left ventricle; P, pulmonary valve; RV, right ventricle; T, tricuspid valve. Reprinted from Sheehan and Redington [23] with permission from BMJ Publishing Group, Ltd. Copyright © 2008, BMJ Publishing Group, Ltd

**Fig. 5:**
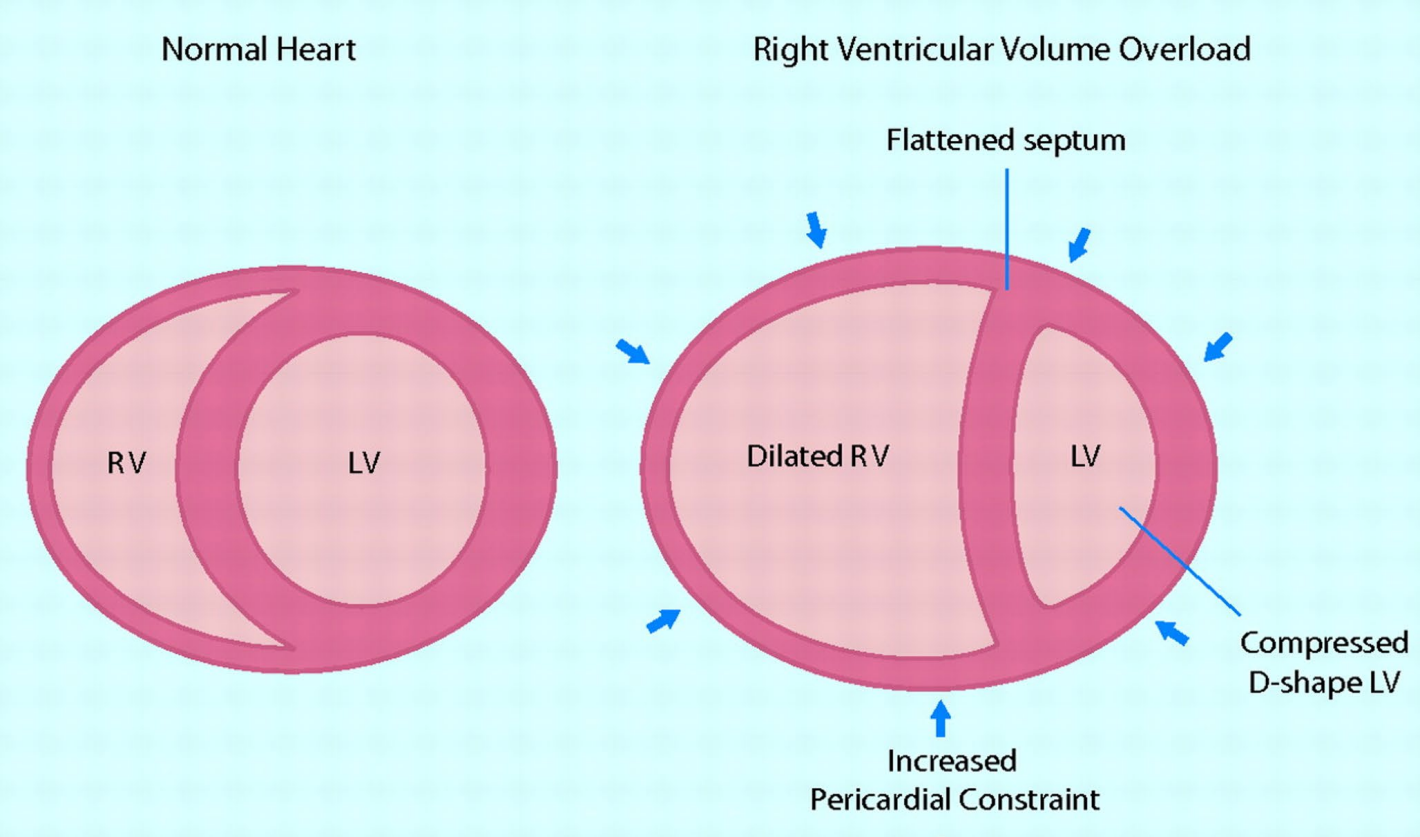
The effects of RV volume overload on cardiac interactions. Right ventricular volume overload causes RV cavity dilation, which flattens the interventricular septum and increases pericardial constraint, ultimately compressing the left ventricular cavity. This leads to impaired LV diastolic filling, reduced LV distensibility and elastance, and decreased cardiac output. Reprinted with permission from Haddad F. et al. [22]

As RV dysfunction progresses to RVF, the RV chamber distorts from its normal triangular shape to a more spherical one (Fig. 4). This structural distortion leads to further deleterious effects. In the TG model, the ascendant apical loop is where the RV and LV share the myocytes of IVS. As the RV dilates, the IVS shifts from right to left, compressing the LV and limiting LV diastolic filling (Fig. 5). The flattening of the septum results in decreased traction for the RV free wall to contract radially leading to further impaired contractility by limiting RV torsional passive impelling contraction [22]. The distortion of the RV structure also results in delayed RV systole leading to further decrease in LV filling which, in turn leads to a decrease in CO, hypotension and decreased coronary perfusion pressure which may lead to a decrease in cardiac contractility. Additionally, RV distention results in increased pericardial constraint leading to compression of the right marginal artery resulting in relative coronary ischemia leading to further impairment of cardiac contractility [16].

## Precipitants of RVF in the MICU

The precipitants of RVF in the critical care setting can be subdivided into three underlying pathophysiological dysfunction subtypes: Preload, Afterload, and Contractility. The common causes for Preload dysfunction include acute renal failure (ARF), acute valvular insufficiency and patent foramen ovale. Common causes of Afterload dysfunction include pulmonary embolism, hypoxia, acidosis, and PPV. Contractility dysfunction most commonly results from RV myocardial ischemia, myocarditis, and arrhythmias [3]. Etiologies can be further subdivided into acute and acute-on-chronic given the differences in presentation and management. This distinction is important because the approach to the underlying dysfunction is fundamentally different. While an obstructive PE and RV myocardial ischemia can present suddenly in a previously healthy individual, RVF secondary to complications of preexisting left heart disease, chronic lung disease or idiopathic pulmonary hypertension can present as a slower deterioration over days to weeks. The approach to the management of these two entities is fundamentally different, where acute RVF etiologies can be targeted by thrombolysis or invasive procedures, such as thrombectomy or percutaneous coronary intervention (PCI) and lead to radical improvement upon resolution. While acute-on-chronic entities require strategic intensivist-driven interventions such as diuresis, ultrafiltration and inotropy. In critically ill patients, it is not uncommon to see overlap of these entities. In those patients, the clinician is required to employ multiple strategies to successfully treat the patient.

## Clinical Manifestations in the MICU

The underlying pathophysiology of RVF is comprised of two cardinal mechanisms, venous congestion and ischemia from reduced CO. Combined, these two pathological processes create a multiplier effect with devastating consequences to the highly vascularized abdominal organs connected in tandem. While reduced CO has been historically the focus of the pathophysiological phenomena surrounding RVF, the last two decades have seen increasing attention given to the venous congestion manifestations of increased filling pressures.

### Cardiohepatic Syndrome

Cardiohepatic Syndrome (CHS) describes a bidirectional relationship between the liver and the heart in a way that one failing organ potentiates maladaptive processes leading to failure of the other [24]. The mere presence of CHS portends poor prognosis in patients with tricuspid regurgitation (TR) treated with transcatheter valve repair [25]. Type 1 CHS represents ischemia-induced hypoxic injury from reduced CO and hypotension (“Shock Liver”), while type 2 CHS represents what is traditionally referred to as “Congestive Hepatopathy”, where elevated central venous pressure (CVP) results in retrograde pressure leading to hepatic venous congestion, bile duct compression and eventually impairment of hepatic synthetic function. Whereas CHS type 1 is most encountered in acute RVF, CHS type 2 is encountered in patients with underlying chronic components leading to parenchymal liver congestion. However, often there is significant overlap between the two types. Megalla et al. found that RV diastolic pressure higher than 16 mmHg and RVF etiology other than LV failure were associated with the development of type 2 CHS, while variables such as LVEF, TR, RVSP and CO did not differentiate between patients with or without type 2 CHS [26]. Patient presentation may include right upper quadrant (RUQ) abdominal pain and encephalopathy combined with physical findings such as hepatomegaly, ascites, hepatojugular reflux and jaundice as a late finding. Significant laboratory abnormalities include hyperbilirubinemia proportional to the elevation in right sided pressures [26]. Aminotransferase elevations are usually modest in the early stages of RVF, and the finding of high levels in the setting of hypotension is suggestive of concomitant ischemic hepatitis.

### Portopulmonary Hypertension

Portopulmonary Hypertension (PPHTN) is PAH associated with portal hypertension, though the exact mechanism is unknown. Of the three theories proposed for the underlying mechanism, the most probable is of an unknown humoral mediator causing pulmonary vasoconstrictive and vasodilatory imbalance. Other theories include genetic predisposition, inflammation, and thromboembolic events. Interestingly, the development of PPHTN does not correlate with the severity of liver dysfunction [27]. PPHTN is the result of pathological changes in the pulmonary vasculature indistinguishable from other PAH etiologies. These changes result in decreased pulmonary compliance which can lead to hypercapnic respiratory failure in patients with borderline clearance of CO_2_. As mentioned previously, hypercapnia leads to worsening acidosis, which in turn results in further increase in PVR and RV afterload. Treatment with pulmonary vasodilators is very effective and allows patients to proceed to liver transplantation [27].

### Cardiointestinal Syndrome

The gastrointestinal manifestations of RVF are one of the least researched, understood and recognized areas in critical care, despite the associated high morbidity and mortality. Alterations in intestinal morphology, permeability, and absorption are some of the common GI manifestations of RVF associated with significant morbidity and mortality [28]. Under normal circumstances, the splanchnic vasculature contains 25% of the human blood volume [29]. Its flexible capacitance plays an important role in regulating normal RV preload by adjusting to perfusion pressure and autotransfusion when necessary; facilitated through elastic recoil and selective sympathetic activation of vasoconstrictive and vasodilatory receptors. Normally, the RV maintains a low CVP, permitting venous return at a low mean systemic filling pressure (MSFP). While venous return can be augmented by raising CVP, this requires an increase in MSFP and risks venous and visceral congestion. As central venous congestion is transmitted back to the splanchnic circuit, capacitance increases, accommodating a larger share of the effective vascular volume (EVV). To counter the resulting decrease in EVV, neurohormonal activation triggers compensatory vasoconstriction of capacitance veins via α-receptors and hepatic venodilation via β2-receptors. Once splanchnic congestion approaches maximal capacity, central venous pressures rise sharply, followed closely by cardiac filling pressures [30]. The low flow state resulting from decreased CO, splanchnic congestion and vasoconstriction leads to shunting of oxygenated blood in the villus base away from its tip, leading to increased enterocyte hypoxic injury [31]. Hypoxic injury of villus enterocytes leads to intestinal barrier dysfunction and increased permeability. Consequently, endotoxins and lipopolysaccharides produced by gram-negative bacteria enter the circulation, triggering systemic vasodilation and the release of inflammatory cytokines, resulting in cardiomyocyte impairment. This impairment is further potentiated by decreased cytokine clearance from kidney dysfunction [32]. It is important to note that current clinical evidence does not support routine antibiotic use for prophylaxis or treatment of suspected bacterial translocation in RVF. In addition, villus ischemia results in acidosis known to increase intestinal Na reabsorption followed by water, thereby increasing volume overload even further [31].

As visceral edema contributes to impaired intestinal barrier and bacterial translocation-induced endotoxin release, an immune response is triggered by the lymphoid tissue abundant in the GI tract. This response is characterized by cytokine release, which in turn induces release of cortisol, norepinephrine and epinephrine. In contrast to the splanchnic vasoconstriction induced by therapeutic vasopressors, which increases venous tone, stress volume, and MSFP to enhance venous return and reduce visceral congestion (as in hepatorenal syndrome), this endogenous response expands EVV at the cost of worsened congestion [33]. Increased hydrostatic pressure in the splanchnic circulation results in increased filtration demands on the lymphatic system. Once its maximal clearance rate is exceeded, abdominal lymphatic flow decelerates, leading to accumulation of protein-rich edema, inducing a further increase in visceral edema and development of abdominal ascites. Ascites contributes to increased intraabdominal pressures by compressing renal vasculature resulting in limited renal perfusion, clearance and urine flow. In addition, intestinal wall edema and hypoperfusion from congestion-induced reduced blood flow result in increased intestinal epithelial permeability leading to protein loss into the lumen [28].

The resultant edema further exacerbates the intestinal rate of absorption leading to nutritional deficiency and decreased rate of oral medication absorbance necessitating transition to IV medications. Nutritional deficiency is further exacerbated by hypoproteinemia from protein-losing gastropathy, decreased appetite, nausea, and vomiting. A study of symptoms experienced by hospice patients with HF suggests that the most common symptoms they present with are poor appetite (38%), followed by nausea (20%) and vomiting (10%) [33].

### Ischemic Colitis

Ischemic Colitis (IC) is a frequent finding in patients with heart failure and commonly occurs in the presence of inferior mesenteric branches atherosclerosis. A Recent systematic review found that patients with HF have a 3.4-fold higher risk of developing IC [34]. The most common mechanisms contributing to IC are hypoperfusion from reduced CO and embolic disease. Intestinal injuries can range from inflammation to full necrosis and bleeding, which in the setting of the stressed intestinal mucosa from RVF can lead to accelerated pathology and rapid deterioration [35].

### Kidney Failure and Cardiorenal Syndrome

The presence of renal dysfunction is a long-established indicator of poor outcomes in RVF patients [36]. One of the most challenging aspects of successful treatment of RVF is achieving negative fluid balance while concurrently improving kidney function. Historically, worsening kidney function in the setting of RVF was thought to be a derivative of decreased perfusion and over-diuresis. More recently, the importance of venous congestion has been recognized as a major contributor to this maladaptive process. Mullens et al. found that worsening renal function in RVF patients correlated with increasing CVP rather than measures of perfusion or RV function [37]. Increasing right-sided filling pressures result in central venous congestion transmitted back to the renal vein and decreasing GFR. This is further exacerbated by the accompanying decrease in CO which in turn leads to activation of RAAS, cascading further to worsening kidney dysfunction, increased diuretic resistance, and decreased UOP. Worsening kidney function may provide an early indication of worsening RVF as both sides of the heart fail to maintain adequate CO to provide effective renal perfusion and avoid congestion. The mechanisms underlying kidney dysfunction intersect where low CO potentiates RAAS activation leading to sympathetic mediated splanchnic vasoconstriction resulting in increased EVV, while total body volume remains constant, exacerbating venous congestion [38].

### Pleural Effusions

Mounting evidence suggests that the presence of pleural effusions in RVF patients is associated with increased mortality. This is not surprising as the appearance of pleural effusions signifies more advanced disease with more systemic involvement. Furthermore, pleural effusions impair lung expansion, potentially leading to hypoxia and respiratory compromise. Increased venous congestion and systemic venous pressure can lead to increased hydrostatic pressure in systemic capillaries, leading to fluid leakage into the pleural cavity. This process is compounded by impaired pleural lymphatic drainage and increased pulmonary venous pressures promoting pleural fluid accumulation. In patients with cardiohepatic syndrome, reduced albumin synthesis lowers oncotic pressure, promoting fluid extravasation into the pleural space. These effusions are typically transudative, characterized by low protein content, as they primarily result from increased congestion and low protein. Thoracentesis for large or symptomatic pleural effusions can lead to dramatic improvement in symptoms [39].

## Evaluation and Diagnosis

The initial evaluation plays a key role in diagnosing right ventricular failure (RVF), assessing its severity, and identifying potential etiologies. A thorough history provides critical context for RVF’s nonspecific presentation, including symptoms like dyspnea, fatigue, diaphoresis, early satiety, abdominal pain, and lower extremity edema, alongside vital signs such as tachycardia, hypotension, or narrowed pulse pressure.

Despite the increasing reliance on advanced diagnostics over traditional history-taking and physical examination, the physical exam remains essential in RVF diagnosis, particularly given its nonspecific symptoms and findings. A targeted, efficient physical exam, integrated with modern technological modalities, optimizes diagnostic accuracy. Specifically, cardiac examination may reveal a loud pulmonic second sound or tricuspid regurgitation murmur, while clear lung fields on auscultation help rule out left ventricular etiology, balancing timeliness with essential insights.

One of the primary objectives of the physical examination in patients with RVF is volume status evaluation, which can be accomplished by checking for the presence of peripheral edema, jugular venous distention, and HJR. In patients with left sided heart failure, the presence of elevated jugular venous pressure was associated with increased HF hospitalization and death [40]. The abdominal exam is also important to identify ascites and increased abdominal pressure, which may impact abdominal circulation and UOP. Assessing volume status is particularly important in patients experiencing hypotension, renal dysfunction, and hypoxia given the rapid deterioration they may experience if not addressed promptly. POCUS has emerged as a valuable tool for determining volume status based on key evaluations of ascites, pericardial effusion, pleural effusions, pulmonary edema, and peripheral edema.

POCUS, particularly via a transthoracic approach, is a valuable tool for assessing right ventricular (RV) dysfunction, given that the RV is the most anterior cardiac structure. However, imaging the RV can be challenging to obtain and reproduce due to its retrosternal position and unique geometrical structure, where the inflow and outflow tracts lie nearly in the same plane. This complexity makes RV imaging harder to obtain and reproduce compared to LV evaluations, and it can also be operator dependent [41]. To ensure a reliable and comprehensive evaluation of RV function, multiple measurements and views are essential [42]. Additionally, developing a standardized approach to the bedside echocardiogram is critical to avoid overlooking key details, despite the added challenges posed by the RV’s distinct anatomy.

Pulmonary artery catheterization (PAC), while useful in select patient populations, is associated with the complications of central venous access as well as the potentially catastrophic complications of cardiac arrhythmias, RV puncture, and pulmonary artery rupture [43]. It also requires additional equipment as well as a skillful clinical team that may not be available in all ICUs, particularly non-cardiac ICUs. It has been argued that decreased familiarity may lead to inaccurate measurements or other complications, including increasing tricuspid regurgitation. The passage of a PAC through the tricuspid valve has been shown to exacerbate TR, particularly in patients with pre-existing TR, by mechanically interfering with coaptation of the tricuspid leaflets [44]. While the ESCAPE trial found no mortality benefit for the routine PAC use in heart failure [45], the exacerbation of TR due to PAC-induced leaflet mal-coaptation may introduce a bias by making patients appear to have worse hemodynamics on introduction and artificially improved outcomes from removal of the mechanical disturbance potentially skewing results in favor of PAC usage.

### Echocardiographic and Ultrasound Assessment in RVF

**Fig. 6:**
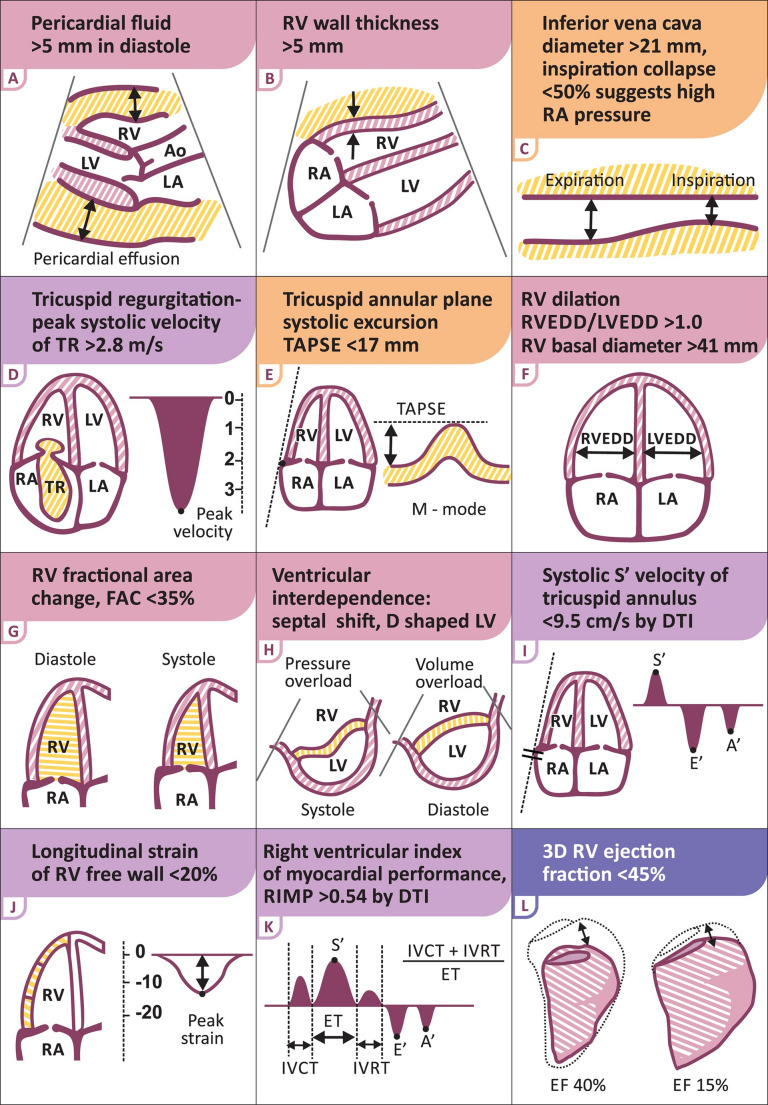
Graphic representation of POCUS parameters for RVF assessment. Adapted by permission from Harjola VP et al. [61]

The advent of echocardiography has allowed for non-invasive estimations of important cardiovascular hemodynamic parameters such as cardiac output [46], right ventricular pressure, and pulmonary artery pressure [47]. In patients with PAH, invasive measurements of PASP deterioration correlated well with non-invasive echocardiographic measurements in a prospective study [48]. While further prospective studies are needed to confirm its applicability in all scenarios, POCUS echo is an expedient, cheap, safe and effective way to provide hemodynamic estimates in a dynamic setting without additional equipment, training, and procedural risk. While there are no head-to-head studies comparing PAC to POCUS to date, POCUS is the preferred first-line option for diagnosis of RVF, while PAC should be reserved for the select patients requiring detailed hemodynamic profiling or continuous monitoring. Commonly used parameters and reference values for RVF assessment are graphically depicted in Fig. 6.

### Volume vs. Pressure Overload Assessment

In hypotensive patients with RVF, RV stress often reflects a combination of pressure and volume overload rather than pure forms of either, with mixed etiologies common in the ICU. While pressure overload may predominate in systole and volume overload in diastole, these patterns primarily indicate the severity of RV decompensation. Ventricular interdependence, marked by septal shift and D-shaped LV in systole on parasternal short-axis view (Fig. 6—panel H), suggests significant pressure overload. The passive leg raise test may assess fluid responsiveness, but current evidence is limited, and its routine use requires further validation.

### Venous Excess Ultrasound (VExUS) Score

Although IVC dilation and absent respiratory variations are widely used POCUS parameters to assess fluid-responsiveness and clinically significant venous congestion in the ICU, evidence shows the IVC dilation alone has poor diagnostic accuracy for these purposes [49]. The VExUS score (Grades 0–4) integrates pulse-wave Doppler measurement of the hepatic, portal, and renal veins to give an objective, quantifiable, real-time assessment of the severity of systemic and multiple end-organ venous congestion. Higher scores correlate with elevated right atrial pressure (RAP), offering a more sensitive measure than IVC assessments alone [50]. As a result, it can assist in predicting organ injury early, thereby helping clinicians tailor fluid-management therapies with more precision and individualized goals. In addition, high VExUS scores are associated with increased in-hospital mortality, longer ICU stays, and a higher need for renal replacement therapy [51, 52]. While POCUS is non-invasive, inexpensive and widely available, complete VExUS acquisition remains technically challenging and operator-dependent, as evidenced by high inter-rater reliability and reproducibility [53]. Success rates are often reduced in obese patients or those with poor acoustic windows. Furthermore, arrhythmias (particularly atrial fibrillation), ventricular pacing, and conditions that obstruct venous return (such as intra-abdominal hypertension or venous thrombosis) can significantly distort hepatic, portal, and renal venous Doppler patterns, thereby limiting interpretability [54].

### Right Ventricular Function Metrics

(E) Tricuspid Annular Plane Systolic Excursion (TAPSE): TAPSE measures the systolic distance shortening between the apex and the infundibulum reflecting the longitudinal shortening of the RV, primarily driven by the coiling helix of the IVS. It is measured in the apical four-chamber view using M-mode at the lateral tricuspid annulus [55]. It reflects longitudinal RV shortening and correlates with other imaging modalities [56]. According to the 2025 ASE guidelines, TAPSE > 1.7 cm indicates normal RV systolic function, with severity graded as mild dysfunction (≤ 1.7 to > 1.3), moderate (≤ 1.3 to > 1.0 cm), and severe (≤ 1.0 cm) for enhanced prognostic precision [57].

(G) RV Fractional Area Change (FAC): Calculated in the four-chamber view by tracing RV area in systole and diastole. FAC > 35% indicates normal function, with severity graded as mild dysfunction (≤ 35% to > 29%), moderate (≤ 29% to > 22%), and severe (≤ 22%) [57]. Though operator-dependent, it is sensitive and predicts mortality in certain conditions [58].

(I) Systolic S’ Velocity: Using Doppler in the four-chamber view, S’ velocity > 9.5 cm/s at the tricuspid annulus signals normal RV systolic function, with severity graded as mild dysfunction (≤ 9.5 cm/s to > 7.2 cm/s) moderate (≤ 7.2 cm/s to > 5.0 cm/s), and severe (≤ 5.0 cm/s) [57].

Right ventricular outflow tract velocity–time integral (RVOT-VTI) is a pulse wave (PW) Doppler derived measure used to assess RV SV and CO. RV SV is calculated as RV SV = [π × (RVOT diameter/2)^2^] × RVOT-VTI (cm^3^). Pulse wave Doppler is measured across the RVOT and distal RVOT diameter (cm) is measured in mid-systole, 1–2 cm below the PV, on a parasternal short view. A RVOT-VTI > 18 cm is normal, with values < 12 cm indicating severely reduced RV output [57].

### RV and RA Structural Assessment

(F) RV Basal Diameter: Measured in the four-chamber apical view at the RV’s widest point, a diameter > 41 mm suggests RV dilation, though geometric distortion in RVF may affect accuracy [59].

(F) RVEDD/LVEDD Ratio: A ratio < 1.0, measured at end-diastole in the RV base or mid-ventricle, marks RV failure [48], but measurements are prone to error [60].

(B) RV Wall Thickness: Assessed in the subcostal view during diastole, thickness > 5 mm indicates RV hypertrophy, suggesting chronic pressure overload or infiltrative disease in the absence of other pathology [61].

(C) Right Atrial Pressure (RAP) Estimation: IVC diameter > 21 mm with < 50% diastolic collapse, or (D) peak tricuspid regurgitation (TR) velocity > 2.8 m/s, indicates elevated RAP.

### Pericardial and Coronary Sinus Evaluation

(A) Pericardial Fluid: Best evaluated in the subxiphoid view during diastole when intracardiac pressures are low. Fluid measuring > 5 mm (approximately > 100 ml) raises concern for tamponade in RVF [62], warranting further assessment for pericardiocentesis.

Coronary Sinus Dilation: Observed in parasternal or apical two-chamber views [63], a diameter > 1 cm (normal 0.6–1.6 cm) may indicate RV pressure overload, correlating with RV dysfunction [64].

### Pulmonary Artery Systolic Pressure (PASP) and Coupling

(D) PASP Estimation: In the four-chamber view, use continuous-wave Doppler to measure TR velocity, combined with IVC-based RAP estimation. PASP = RAP + 4 × (TR velocity)^2^, though TR jet accuracy is critical to avoid errors.

(D, E) TAPSE/PASP Ratio: This ratio assesses the relationship between RV contractility and afterload, serving as an indicator of RV function and pulmonary artery (RV-PA) coupling. RV-PA uncoupling reflects impaired RV adaptation to increased load [53]. A recent single-center study demonstrated that patients with acute PE who exhibit severe RV-PA uncoupling ratios (< 0.31) are more likely to have severe disease. TAPSE/PASP offers superior risk stratification and treatment guidance, better than traditional scoring systems, particularly in intermediate-high risk patients [57, 65].

### Bubble Study for Shunting

A bubble study (agitated saline contrast echocardiography) showing microbubbles crossing from the right to the left atrium within three cardiac cycles confirms right-to-left shunting across a patent foramen ovale (PFO), which may cause persistent hypoxemia in worsening RVF [66].

### Biochemical Markers

Biochemical markers play an important role in assessing severity and predicting prognosis, though none are specific to RVF [67]. Elevated troponin I and T, indicative of RV ischemia, are highly sensitive and associated with increased mortality risk in acute RVF, particularly in settings like pulmonary embolism (PE) and COVID-19, aiding in triage and prognosis [68, 69]. Markers of ventricular wall tension such as B-type natriuretic peptide (BNP) and N-terminal pro-BNP (NT-proBNP), correlate with increased ventricular pressures [70, 71] and are negatively correlated with RV ejection fraction in pulmonary arterial hypertension (PAH) and chronic thromboembolic pulmonary hypertension (CTEPH) [72]. However, low BNP levels may be seen in heart failure with preserved ejection fraction (HFpEF) and obesity [73]. Hyponatremia, driven by neurohormonal activation (e.g., vasopressin, angiotensin II), reduced cardiac output, and impaired renal function, is more prevalent in severe RVF and predicts mortality [74, 75], as RV dysfunction exacerbates water retention and systemic congestion [76] leading to worsening hyponatremia. Creatinine elevation reflects a complex interplay in RVF, where worsening renal function increases sodium and fluid retention, elevating right atrial pressure (RAP) [77] while RV dysfunction contributes to renal hypoperfusion through venous congestion, reduced cardiac output, and neurohormonal changes [78]. C-reactive protein (CRP) is linked to RV dysfunction and PE-related mortality [79] as well as post-left ventricular assist device (LVAD) RV failure [80], suggesting additional inflammatory mechanisms beyond RVF alone. Finally, in a small study of persistent RVF post-revascularization, lactate reduction following Impella RP placement highlighted its potential as a marker of improved RV function [81]. Like elevated creatinine, RVF may also lead to liver dysfunction through both congestion and reduced perfusion. LFTs (AST/ALT and direct/indirect bilirubin) may be elevated in RVF due to elevated RAP causing hepatic congestion as well as from decreased cardiac output. Over time, persistent reduced perfusion and congestion may lead to fibrosis and cirrhosis [82]. These biomarkers collectively enhance the diagnostic and prognostic evaluation of RVF, guiding clinical management.

## Management of RVF in the MICU

In managing RVF in the MICU, priority should be given to addressing potentially reversible causes. This includes minimizing the use of common ICU medications and treatments that may exacerbate RVF, such as alpha-agonists, negative inotropes, and hypotension-causing agents like Dexmedetomidine. After mitigating reversible etiologies, the treatment strategy for acute RVF in critically ill patients should focus on three core mechanisms: (1) optimizing RV preload to reduce excessive volume and prevent RV dilation; (2) Reducing afterload to alleviate RV strain; and (3) enhancing contractility to improve RV function.

### RV Preload Optimization

Volume maintenance is one of the most impactful ways to improve hemodynamics in RVF. RV dilation results in anatomical distortions leading to decreased contractility of the RV itself and shifting the IVS leftward, thereby decreasing LV size and CO. A simple strategy to implement early in the course is intravenous fluids (IVF) stewardship with special attention given to stopping continuous IVFs and increasing the concentration of pharmacotherapeutics. While there has been a traditional teaching that acute RVF should be treated first with a trial of small IVF bolus [22], this practice should be reserved for patients with clear evidence of hypovolemia, septic shock or acute RV infarction, where insufficient preload leads to decreased RV output. Studies have shown that volume loading patients with increased PVR results in a counter effect of decreased LV diastolic volume [83]. In hypervolemic patients, improved perfusion can be achieved with vasopressors and inotropy while avoiding the adverse effects of increased preload and deterioration into a detrimental spiral.

#### Diuretics

Acute RVF often results in decreased renal blood flow leading to neurohormonal activation of the renin–angiotensin–aldosterone system (RAAS). This leads to increased sodium reabsorption, contributing to increased water reabsorption and loop diuretic resistance. The use of an early aggressive diuresis strategy is imperative for successful RVF treatment, as inadequate diuresis is associated with worse outcomes in acute decompensated HF patients with fluid overload. While diuresis is one of the most important treatments for RVF; it is important to view it also as a preventive measure against further deterioration.

There is robust evidence from multiple clinical trials, observational studies and meta-analyses consistently demonstrating that inadequate diuresis is associated with worse outcomes including increased mortality [84–87]. It is advisable to initiate treatment with high-dose loop diuretics to exceed the diuretic threshold, particularly in patients with decreased creatinine clearance (CrCl), who require higher doses to achieve adequate diuretic concentrations at the site of action [88, 89]. In the case of Furosemide, increasing the diuresis effect can be achieved by more frequent dosing every six hours once the diuretic threshold is established, as further dose escalation does not enhance the effect. Bolus dosing is preferred over continuous loop diuretic infusion due to its higher likelihood of reaching the diuretic threshold faster. Dosing should consider diuretic naivety, home dose, and available data such as spot urine sodium (UNa) or urine output (UOP) since presentation. A baseline spot UNa before diuretic administration can guide dosing and additional testing can be used to assess response, with UNa < 50 mmol/L indicating inadequate response, requiring dose increase [90]. In a prospective cohort study acute HF patients with low UNa excretion in the first six hours after initiation of loop diuretic therapy were found to have lower UOP and higher all-cause mortality [91]. It can be argued that spot Una is a more valuable parameter than UOP for assessing diuresis, as it provides earlier insights into diuretic response, enabling clinicians to adjust treatment regimens more rapidly during critical periods.

#### Diuresis Targets

In severe acute RVF, diuresis targets should aim for 100–150 mL/hour (2–3 L/day) not to exceed 150 mL/hour to balance efficacy and safety, and CVP < 8 cm H_2_O [2, 37, 85, 90]. CVP reduction is associated with higher CO and improves 28-day prognosis in patients with circulatory shock [92]. The routine monitoring of daily weights and strict Ins and Outs has been one of the cornerstones of diuretic treatment response monitoring; however, recent studies found those to be poor measures due to being inaccurate and challenging to obtain in a timely fashion [93]. It is advisable to assess UOP goal and electrolytes every six hours in the initial 24 h, then every 12 h or assess natriuresis with spot urine sodium targeting goal of > 50–70 mmol/L after 2 h from diuretic regimen initiation [89]. It is not advisable to use oral diuretics given the high likelihood of decreased GI reabsorption from hypoperfusion or congestion of splanchnic circulation [94]. In patients with diuretic resistance and hypoproteinemia (from cardiointestinal syndrome), some studies suggest that coadministration of albumin can be beneficial [95].

Close monitoring of diuresis response, through hourly UOP recording or spot UNa, is essential for achieving negative fluid balance in a timely manner. A recent meta-analysis showed that high UNa after diuretic administration is associated with higher UOP, shorter hospital stays, and lower mortality odds [96]. The ENACT-HF study, a prospective, open-label trial, demonstrated that a protocolized diuresis regimen-guided natriuresis was superior to each institution’s standard of care. Patients on a diuresis protocol were found to have 64% higher natriuresis in the first day of treatment with sustained higher natriuresis and diuresis in the first two days, as well as one day shorter length of stay. The difference was even greater for patients with GFR < 49 or on prior high oral loop diuretic maintenance regimen [97]. The presence of systemic hypotension should not deter aggressive diuresis in hypervolemic patients; vasopressors can temporarily support systemic perfusion until RV hemodynamics improve. The widespread assumption that elevated creatinine and AKI invariably indicate tubular injury should be reconsidered, as multiple studies suggest this is not the case, but merely a reduction in GFR [98]. Studies like DOSE have shown that higher doses (e.g., 2.5 × home dose, up to 100 mg) are safe and efficacious [84], while other studies evaluating kidney function post-diuresis in patients with elevated CrCl further support these strategies. Ahmad et al. showed that while patients receiving high-dose loop diuretics experienced mild to moderate decrease in eGFR, this was not associated with elevations of tubular injury biomarkers, increased mortality or rehospitalization and possibly contributed to premature cessation of decongestive treatment. Another noted result was that changes in metrics of diuresis such as UOP and UNa were not linked to measures of kidney function such as creatinine and cystatin-C [99]. Further, in patients with Intra-abdominal pressure (IAP) > 8 mmHg diuresis also helps improving kidney function via decreasing abdominal hypertension [100]. Intraabdominal hypertension develops when IAP exceeds 8 mmHg and results in further organ dysfunction and most importantly decreased renal function, especially in patients who develop compartment syndrome. In patients with severe IAP, it is advisable to perform routine measurement of IAP via bladder pressure and paracentesis for frank ascites to improve kidney function [101]. In refractory cases, surgical management may be necessary.

#### Adjunctive Therapy

Patients with diuretic resistance, severe volume overload and electrolyte derangements are likely to benefit greatly from combination diuretic therapy. While minimizing electrolytes disturbances is important, the removal of high volume of fluid expediently is of most importance in the initial stages of RVF treatment; therefore, the combination therapy contributing most to that should be preferred, while monitoring electrolyte levels closely.

In cases of severe RVF, non-respondents and cases where cardiorenal syndrome is suspected, early initiation of distal diuretics treatment is advised. A distal diuretic such as a thiazide, can be used in patients with suspected loop diuretic resistance-induced epithelial cell or distal tubular hypertrophy from chronic loop diuretic use [102]. In patients with hyperkalemia, initiation of thiazide or aldosterone antagonists such as Amiloride or Spironolactone may increase diuresis yield.

Although the ADVOR trial enrolled predominantly left-sided or biventricular heart failure, Acetazolamide, a carbonic anhydrase inhibitor (CAI) that blocks the proximal convoluted tubular absorption of sodium, in combination with loop diuretics, has emerged as an effective component of sequential nephron blockade in diuretic-resistant congestion [103]. CAIs should be used in cases of hypochloremic metabolic alkalosis, which may result from aggressive diuresis. However, given that most severe RVF patients are acidemic, caution should be used as CAIs cause normal AGMA.

Sodium bicarbonate enhances diuretic responsiveness by urine alkalinization and correcting metabolic acidosis, thereby improving myocardial contractility and catecholamine responsiveness. However, the high sodium load associated with its administration may increase fluid retention and adversely affect RV preload. Additionally, in patients with concurrent respiratory failure, it may exacerbate hypercapnia by increasing CO_2_ production, potentially leading to an increase in PVR. 

#### Ultrafiltration

Early ultrafiltration (UF) for volume optimization can be significantly beneficial to RVF patients with volume overload, low urine output, and inadequate response to diuretics. While resource-intensive, prompt recognition and commitment to initiation of UF is essential for successful treatment, preventing a downward spiral that becomes challenging to reverse once established. In cases of severe interstitial edema, UF proves more efficient as it allows for the removal of more sodium for the same amount of fluid compared to diuretics [104]. In severe cases, it is advisable to initiate early UF within six hours of determining an inadequate response to a high-dose combination diuretic regimen or failure to achieve net negative fluid balance within the first 24 h of diuresis. As with diuresis targets, the UF goal target of optimal fluid status should be guided by CVP. CVP should be reduced to below 15 mmHg (goal 8–12 mmHg), while avoiding hypovolemia. Caution should be exercised in preload dependent patients, as rapid fluid removal can reduce venous return and result in a decrease in cardiac output. Therefore, avoidance of excessive UF rates (> 200–300 mL/h) is advised in the early stages with titration up as tolerated. It is often beneficial to temporarily withhold or drastically reduce loop diuretic therapy for 48–72 h during active UF, to interrupt the adaptive mechanisms of diuretic resistance, thereby restoring diuretic responsiveness once UF is stopped [105].

### RV Afterload Reduction

While preload management is important in preventing devolvement into a vicious cycle, reducing RV afterload can lead to rapid and dramatic improvement in RV function. Elevated afterload, frequently encountered in patients with preexisting pulmonary vascular diseases, can be treated by lowering pulmonary pressures through thrombectomy, thrombolysis, correction of acidosis, hypoxia and hypercapnia via optimizing ventilation, and decreasing lung congestion through diuresis. In this group, selective pulmonary vasodilators such as inhaled nitric oxide (iNO) and prostacyclin analogs allow for PVR reduction without causing systemic hypotension or worsening VQ mismatch [106]. It has been shown that iNO can enhance RVEF, reduce end diastolic volume, and improve pulmonary hemodynamics and oxygenation in RVF patients [107, 108]. In patients with RVF driven by left-sided heart failure with adequate blood pressure, decreasing LV afterload with systemic vasodilators may also reduce PVR and expedite the achievement of diuresis goals by improving renal perfusion and reducing preload. However, caution should be used when using pulmonary vasodilators in patients with left ventricular dysfunction and high left-heart resistive loads, as increasing the flow through the pulmonary circuit may worsen pulmonary vascular congestion by allowing for greater forward flow into an already weakened and overloaded LV [109]. Ongoing monitoring via POCUS and PAC can aid in tailoring therapy, avoiding complications, and allow for timely escalation to mechanical circulatory support (MCS) in refractory cases.

### Enhancing RV Contractility

RV contractility is impaired in acute RVF due to RV free wall myocyte mechanical stretching, impaired oxygenation and cellular metabolic derangements. This can be improved through several targeted strategies, tailored to the underlying etiology, with improving LV contractility serving as a critical approach. As mentioned previously, studies suggest that the LV contributes up to 60% of the RVEF via IVS contraction, driven primarily by the helical fibers of the myocardium with significant contribution from the basal loop’s fiber architecture [2, 110]. Inotropic agents such as epinephrine, dobutamine and milrinone can improve RV function primarily by improving LV function through enhancing IVS contractility, rather than improving the function of an already distended RV free wall.

The choice of inotropic agent depends on several factors such as PVR, SVR and CO. Milrinone, a phosphodiesterase-3 inhibitor, is preferred in patients with high PVR and without systemic hypotension as it offers the highest reduction in PVR but causes peripheral vasodilation. Dobutamine, A β1-adrenergic agonist, improves contractility with minimal impact on PVR and minimal reduction of SVR. It is the ideal choice in patients with mild PVR elevations, normal to low SVR and low CO. In contrast, low-dose epinephrine is ideal for patients with low SVR, providing inotropic support without the systemic vasodilation associated with milrinone and dobutamine. However, higher doses of Epinephrine should be avoided in elevated-PVR states, as it worsens pulmonary vasoconstriction. Effective management balances IVS inotropic support with RV afterload reduction, while carefully monitoring for side effects like increased myocardial oxygen demand and tachyarrhythmias [106] (Table 1).

**Table 1:** Hemodynamic effects and the mechanism of action of commonly used inotropic agents used for the management of acute RVF

Inotropic agent	Mechanism/receptor	PVR Effect	SVR effect	Scenario
Milrinone	PDE_3_ Inhibition	Reduction	Reduction	High PVR, normal/high SVR
Dobutamine	α1, β1, β2	Minimal	Reduction	Normal/mild PVR, normal/low SVR
Epinephrine	α1, β1, β2	Minimal (low dose)	Increase	High PVR, low SVR/shock

*PVR* pulmonary vascular resistance, *SVR* systemic vascular resistance, *PDE*_*3*_ phosphodiesterase 3

#### Coronary Perfusion

Optimizing coronary perfusion is critical to ensure adequate myocardial oxygen delivery and maintain CO in RVF. RV dilation and increased wall tension can impair coronary perfusion through mechanical compression exerted by the limited space of the pericardial sac. This effect is particularly pronounced in patients with coronary artery disease, where reduced myocardial oxygen supply may precipitate a critical decline in RV function. In cases of inferior myocardial infarction, timely revascularization can significantly improve outcomes.

Currently, there are no guidelines for the optimal BP targets in RVF, and the MAP target of 65 mmHg has been used universally in different types of shock to support perfusion [106, 111]. Unlike the left, right-sided coronary perfusion takes place during both systole and diastole, requiring optimization of MAP rather than SBP. The interplay between perfusion and congestion is demonstrated well by a retrospective study of pre-capillary PH patients with RVF admitted to an ICU, showing that patients with a dynamic MAP target, based on MAP = 60 + CVP to optimize systemic perfusion pressure (SPP), had lower in-hospital mortality and incidence of AKI compared with patients with static targets of MAP 65 or 70 [112]. Therefore, systemic hypotension should be avoided due to concern for hypoperfusion of the coronary circulation, kidneys, and bowels. When selecting inotropes and pressors, the goal is to optimize SPP by increasing SVR and RV contractility, with minimal increase in PVR, which may worsen RV failure by increasing RV afterload [106]. It is important to note that catecholamines increase myocardial oxygen demand, while vasoconstrictors may compromise microcirculation and tissue perfusion. Therefore, their use should be limited to the lowest effective dose and the shortest duration necessary [113].

#### Choice of Vasopressors

In the management of RVF with hypotension, initial treatment involves vasopressin, which reduces PVR while increasing SVR, thereby supporting SPP and coronary perfusion without exacerbating RV afterload. Low-dose epinephrine can be added to enhance these effects and provide inotropic support, improving CO while maintaining minimal impact on PVR. If hypotension persists, norepinephrine is titrated to achieve a MAP target for optimal SPP with minimal PVR increase, making it a first-line choice for persistent hypotension in RVF. Phenylephrine is not recommended, as it increases PVR, worsening RV afterload and potentially reducing CO. Similarly, methylene blue should be avoided due to its significant increase in PVR, which can further impair RV function. Dopamine and angiotensin II (ATII) are also contraindicated in RVF, as dopamine risks arrhythmias and variable PVR effects, while ATII increases both PVR and SVR, potentially straining the RV [88] (Table 2).

**Table 2:** Hemodynamic effects and the mechanism of action of commonly used vasopressor agents used for the management of acute RVF

Agent	Dose	Receptor/mechanism	PVR	SVR	CO	Notes
Vasopressin	0.01–0.04 units/min IV (fixed dose, not titrated to kg)	V1a, V2, V1b	↓	↑	< – >/↑	Preferred in systemic hypotension; ↓ PVR, maintains higher GFR, urine output
Epinephrine	0.01–0.1 µg/kg/min IV (low-dose for inotropy; higher doses for vasopressor effect)	α1, β1, β2	< – >/↑	↑	↑	Low dose preferred in RVF with hypotension and low SVR; high doses may ↑ PVR
Norepinephrine	0.01–1 µg/kg/min IV (titrated to MAP 60 + CVP)	α1, β1	< – >	↑	< – >/↑	minimal PVR impact, supports coronary perfusion
Phenylephrine	0.1–2 µg/kg/min IV (titrated to MAP)	α1	↑	↑	↓/< – >	AVOID; ↑ PVR worsens RV afterload, may reduce CO
Dopamine	2–20 µg/kg/min IV (low: 2–5 µg/kg/min for inotropy; high: > 10 for vasopressor)	α1, β1, D1, D2	< – >/↑	↑	↑/< – >	AVOID; risk of arrhythmias, variable PVR effects
ATII (Angiotensin II)	1–20 ng/kg/min IV (titrated to MAP)	ATR1, ATR2	↑	↑	< – >/↓	AVOID; ↑ PVR and SVR may strain RV
Methylene Blue	1–2 mg/kg IV bolus	Inhibits NO synthase	↑	↑	↓/< – >	AVOID; causes increased PVR, worsens RV afterload

*PVR* pulmonary vascular resistance, *SVR* systemic vascular resistance, *CO* cardiac output, *GFR* glomerular filtration rate, *MAP* mean arterial pressure, *CVP* central venous pressure, *ATR* angiotensin II type receptor, *NO* nitric oxide, *RV* right ventricle

#### Correction of Myocardial Depression

Of great importance is enhancing myocardial contractility by reversing conditions that directly result in reduction of myocardial contractility. Aggressive treatment of acidosis, hypoxia, and electrolyte imbalances such as hypokalemia and hypomagnesemia can often result in significant improvement in RV hemodynamics.

Rhythm control is another important factor in myocardial contractility. One of the characteristic anatomical changes in RVF is cardiac chamber dilation. Chamber wall stretch can lead to increased rates of both atrial and ventricular arrhythmias. These, in turn, can decrease myocardial contractility by disrupting coordinated contraction, increasing oxygen demand, and impairing coronary perfusion. In patients with RVF and atrial fibrillation (AF), RA emptying fraction is lower compared to patients without AF history [114]. Given the RV’s vulnerability, these effects are amplified, making effective arrhythmia management critical [106]. In the setting of hemodynamic compromise or arrest due to RVF, direct current cardioversion (DCCV) is the preferred initial treatment, as it rapidly restores sinus rhythm and optimizes coordinated ventricular contraction by improving cardiac output. This is in contrast to antiarrhythmic drugs, which may act more slowly and risk further hemodynamic instability. To maintain sinus rhythm post-DCCV, amiodarone is recommended as the first-line antiarrhythmic due to its minimal negative inotropic and chronotropic effects, preserving myocardial contractility and heart rate to support CO. In contrast, negative chronotropic and inotropic agents, such as beta-blockers and calcium channel blockers, are contraindicated because they reduce heart rate and contractility, further compromising cardiac output in an already impaired RV. Digoxin may be considered for rate control, as it offers mild positive inotropic effects that can support RV function; however, concerns about renal toxicity and increased mortality with chronic use limit its role to selected cases in which other rate-control agents are ineffective or contraindicated [115].

### Mechanical Support in RVF

The introduction and evolution of MCS transformed care for refractory RVF. It reflects both technological advancement and deeper understanding of the complex structure–function interactions characterizing RVF physiology [116]. While cardiopulmonary bypass, extracorporeal membrane oxygenation (ECMO), ventricular assist devices (VAD) and intra-aortic balloon pumps (IABP) were available since the 1960’s [117], RV specific support devices were not available until the 1990’s. During this interim period, RVF was not yet recognized as a distinct clinical entity and most MCS efforts focused on LV or biventricular failure. The 1990’s saw the emergence of RV-specific MCS with the introduction of pulsatile right-ventricular assist devices (RVAD) [118]. Used as a bridge to transplant, these devices required complex surgery and large external pumps resulting in high rates of morbidity [119]. The TandemHeart was introduced in the late 1990’s and offered a percutaneous approach rather than surgical. Originally created for left atrial-to-femoral artery bypass, it was adapted to RVF by cannulating the right atrium to the pulmonary artery. The 2000’s saw a shift from pulsatile VADs to continuous flow devices, but this proved more useful for LV failure since the RV is more sensitive to changes in preload and afterload. The ImpellaRP was approved by the FDA in 2017, offering the ability to deliver adequate continuous flow to the PA from the RA percutaneously [2].

Effective Impella hemodynamic support is limited in high PVR conditions not responsive to pulmonary vasodilators and biventricular failure, limiting its use to RV infarction and post-thrombectomy [120, 121]. Additionally, its availability is limited due to its high cost and need for expertise. The ProtekDuo, introduced concurrently with the Impella RP, is generally preferred when prolonged support (> 7–10 days), concomitant oxygenation, early ambulation, or internal jugular access (e.g., in patients with IVC filters or severe obesity) is required [121, 122].

The last decade is characterized by the adoption of the idea of biventricular support with the development of the “Bi- PELLA” combining RV and LV Impellas allowing individual flow adjustment to each ventricle, with studies demonstrating improved survival. Recent advances in ECMO cannulation techniques, smaller more efficient circuits and its widespread availability, established ECMO as the MCS of choice in most cases of RVF. Despite ongoing research and innovation, the complex anatomy and physiology of the RV remain challenging for the development of a commercially viable long-term RVAD to date [2].

### Respiratory Support Strategies

#### Endotracheal Intubation

In the event of hypoxemic respiratory failure in RVF patients, preoxygenation with HFNC is of paramount importance to improve oxygen reserve given the deleterious effects of hypoxia and PPV on RV function and hemodynamics as outlined in the previous sections. Endotracheal intubation of patients with acute RVF should be preferably undertaken by an intensivist who is experienced in both high-risk intubations and the hemodynamic implications of doing so in acute RVF. Some of the dangers of intubation in patients with acute RVF are the derivatives of supine positioning such as lung de-recruitment and upsurge in preload. Other risks stem from the use of induction agents such as propofol leading to decreased contractility and SVR, leading to coronary hypoperfusion resulting in further decrease in contractility which may eventually lead to hemodynamic collapse [123]. Even with successful intubation many dangers await in the minutes post intubation. The application of PPV causes an increase in intrathoracic pressure and increasing downstream pressure on hepatic veins and the IVC, leading to decreasing venous return and preload. These effects, coupled with the lingering effects of induction agents and the application of sedatives and analgesics post intubation, may lead to a delayed circulatory collapse many minutes post intubation. It is imperative to observe the patient closely in the post intubation period, add sedatives and analgesics in a stepwise fashion, and continue all RV off-loading therapies post-intubation. Steps must be taken to ensure proper intravenous access to prevent any interruptions in therapy and to facilitate the rapid application of vasopressor support if needed.

#### Ventilator Management

**Fig. 7:**
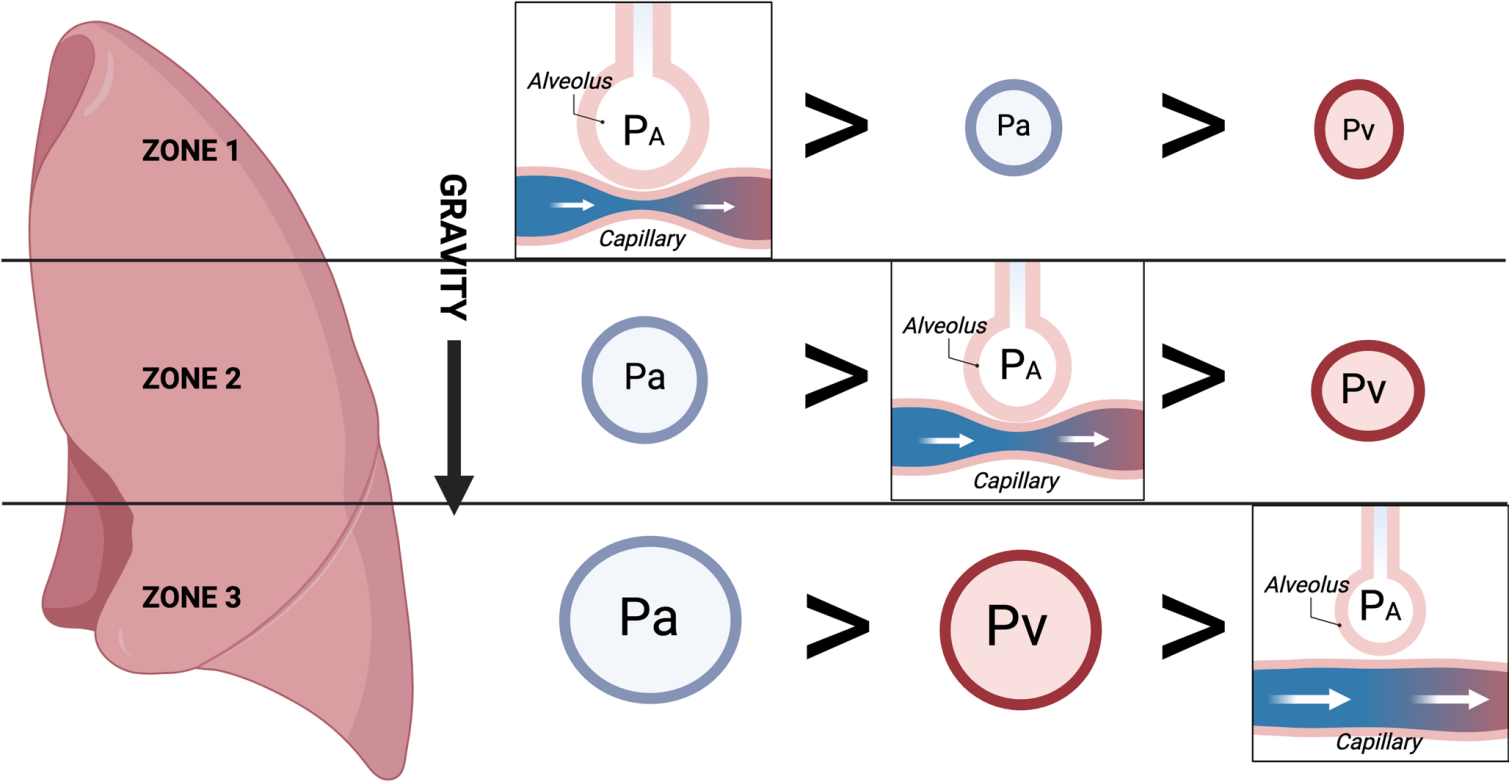
West lung zones under normal conditions illustrating gravity-dependent pulmonary blood flow and ventilation-perfusion dynamics. Zone 1: Alveolar pressure exceeds pulmonary artery and venous pressures creating dead space of ventilated but poorly perfused area. Zone 2: pulmonary artery pressure exceeds alveolar pressure, but alveolar pressure greater than venous pressure, creating a balanced ventilation-perfusion zone. Zone 3: increasing gravity effect results in both arterial and venous pressures exceeding alveolar pressure, resulting in high perfusion relative to ventilation mismatch. When PPV is applied, particularly using high PEEP, alveolar distention may occur. This can lead to compression of extra-alveolar vessels and results in increased PVR, leading to redirection of blood flow into poorly ventilated lung regions and worsening V/Q, resulting in hypoxemia and hypercapnia. P_A_ alveolar pressure, P_a_ pulmonary artery pressure, P_v_ pulmonary vein pressure. Adapted from [17]. Created in BioRender. Dodi, A. (2025) https://BioRender.com/ngngks2

Positive pressure ventilation is used in many critically ill patients and results in increased intrathoracic pressure leading to increased RV afterload [124]. The effects of PPV on RV hemodynamics are best explained by dividing the lung field into three vertical zones (Fig. 7), first conceptualized by West [17]. As hydrostatic pressure increases with gravity, the balance between ventilation and perfusion changes accordingly, whereby under normal conditions the alveolar pressure gradient is relatively similar to that of the arterial and the venous creating an ideal ventilation/perfusion (VQ) balance. In zone 1, where arterial and venous hydrostatic pressures are low compared to the alveolar pressure, the VQ is mismatched in favor of ventilation (this is also where pulmonary vascular capacity is capable of expanding in the event of increased volume to ameliorate the rise in PVR). On the other hand, in Zone 3, both arterial and venous pressures are higher than alveolar pressure, resulting in a VQ mismatch in favor of perfusion. With the introduction of PPV, the pressure relationships in the different lung zones change due to the increase in alveolar pressures. Zone 1 has further decrease in perfusion, tilting the VQ relationship further in favor of ventilation. In addition, Zone 1 becomes less available to accept more volume, resulting in the transmission of retrograde pressure into the RV, resulting in an increase in afterload. An additional effect of PPV is that Zone 2 becomes more like Zone 1 prior to the application of PPV, and Zone 3 becomes the area where VQ is most balanced. In situations of RVF with a large component of fluid overload, the presence of significant pulmonary effusions can be detrimental due to compression atelectasis, resulting in a decrease in functional pulmonary areas in zone 3, but also present as a target for rapid improvement by percutaneous drainage. It is important to note that the effects of PPV are seen in both invasive and non-invasive mechanical ventilation [125].

#### Hypoxia and Acidemia Effects on PVR

Elevated RV afterload plays a pivotal role in the evolution of RVF, and its reduction is often very impactful. Hypoxia exerts significant influence on PVR, where alveolar hypoxia increases PVR to a greater degree compared with pulmonary arterial hypoxia. The pulmonary vasoconstrictive response to hypoxia is often accompanied and further potentiated by acidemia, hypercapnia, and atelectasis [106]. The pH of pulmonary capillary blood often changes in association with O_2_ content, which is particularly common during hypo- or hyperventilation and preexisting lung disease (source). Additional contributors of acidemia exacerbation in RVF result from retrograde hepatic venous congestion impairing citrate metabolism and lactate clearance. Another potential source of increased PVR is pulmonary endothelial injury resulting from multiple blood and platelet transfusions (source).

## Conclusion

Given the interdependence between the heart and lungs, it is of paramount importance that accurate diagnosis and treatment of RVF are achieved in a timely fashion during critical illness. To accomplish that, it is imperative for the intensivist to be well-versed in the basics, as well as the recent advances relevant to the field. From a diagnostic perspective, this includes the use of POCUS for the diagnosis of RVF, its etiology, and assessing its severity. From a therapeutic perspective, intensive and decisive volume removal, when appropriate, and use of inotropy and pulmonary vasodilators when indicated, as well as the use of the various temporary mechanical circulatory devices as a temporizing measure. The significant adverse effects associated with PPV should be taken into consideration early in the course and avoided if possible. The future of RVF will benefit from a stronger RV-specific evidence base, distinct from practices derived from LV failure. Emerging diagnostic practices include molecular imaging to facilitate earlier detection and risk stratification, advanced biomarkers, AI-driven imaging analysis, and non-invasive hemodynamic monitoring devices. Emerging therapeutic advances include use of genetic, molecular and hemodynamic phenotyping to deliver personalized, targeted therapies.

## Data Availability

No datasets were generated or analysed during the current study.
